# Incretin-based therapies

**DOI:** 10.1111/j.1753-0407.2011.00143.x

**Published:** 2012-03

**Authors:** Anthony H Stonehouse, Tamara Darsow, David G Maggs

**Affiliations:** Amylin Pharmaceuticals, Inc.San Diego, California, USA

**Keywords:** glucagon-like peptide-1, incretins, pathophysiology, Type 2 diabetes

## Abstract

Incretin-based therapies have established a foothold in the diabetes armamentarium through the introduction of oral dipeptidyl peptidase-4 inhibitors and the injectable class, the glucagon-like peptide-1 receptor agonists. In 2009, the American Diabetes Association and European Association for the Study of Diabetes authored a revised consensus algorithm for the initiation and adjustment of therapy in Type 2 diabetes (T2D). The revised algorithm accounts for the entry of incretin-based therapies into common clinical practice, especially where control of body weight and hypoglycemia are concerns. The gut-borne incretin hormones have powerful effects on glucose homeostasis, particularly in the postprandial period, when approximately two-thirds of the β-cell response to a given meal is due to the incretin effect. There is also evidence that the incretin effect is attenuated in patients with T2D, whereby the β-cell becomes less responsive to incretin signals. The foundation of incretin-based therapies is to target this previously unrecognized feature of diabetes pathophysiology, resulting in sustained improvements in glycemic control and improved body weight control. In addition, emerging evidence suggests that incretin-based therapies may have a positive impact on inflammation, cardiovascular and hepatic health, sleep, and the central nervous system. In the present article, we discuss the attributes of current and near-future incretin-based therapies.

## Introduction

Type 2 diabetes (T2D) is reaching epidemic proportions in Westernized countries owing to the intersection of excess calorie consumption and reduced physical activity imposed on a genetically prone population. Nearly one in 10 individuals in the US have diabetes and it is projected that currently one in three newly born will eventually develop diabetes (see http://www.cdc.gov/chronicdisease/resources/publications/AAG/ddt.htm, accessed 5 October 2011). Diabetes is diagnosed now in younger people and there is much concerned speculation as to how the health economic consequences of diabetes may manifest over the upcoming decades.

Most newly diagnosed and established patients with T2D are overweight or obese and current treatment algorithms place emphasis on initial lifestyle modification entailing a low-calorie diet with regular exercise in order to improve metabolic state and reduce body weight.^1^ Lifestyle modification alone has a low likelihood of sustained metabolic improvement and T2D is a progressive disease, likely through the inexorable decline in β-cell function, and for this reason there is an increasing need for pharmacological intervention over time. The diabetes armamentarium has well-established therapies, each of the more historic mainstays (insulin, metformin and sulfonylureas) having accumulated between 60 and 90 years of clinical experience, and a number of additional oral agent classes introduced over the past 15 years.

In general, if glycemic goals (HbA1c <7) are not achieved or sustained with lifestyle modification, metformin is strongly positioned as first-line oral therapy, largely because of long experience in the clinic, reproducible glucose-lowering efficacy, well-accepted safety and tolerability profiles, neutral effects on body weight, and low cost. Then, in a stepwise manner, further oral agents are added with the eventual need for insulin, first in the basal long-acting form and then later the use of more intensified insulin regimens. Despite this array of oral agents and advances in insulin delivery, pharmacological intervention often falls short of treatment goals with the price of unwanted side effects, namely weight gain and hypoglycemia.

Incretin-based therapies were developed after the observation during the 1960s of an incretin signal originating from the gut that augmented the β-cell response to caloric intake^2^ and the later discovery that the gut hormones glucagon-like peptide-1 (GLP-1) and glucose-dependent insulinotropic polypeptide (GIP) play a central role in this physiological response.^3^–^7^ It is evident that this physiological response is mostly relevant in the postprandial period; however, rodent data have also shown that GLP-1 may play a tonic role in regulating fasting plasma glucose. The incretin response is thought to be defective in T2D, manifesting in part with a complete lack of β-cell responsiveness to exogenously administered GIP.^6^,^8^ The β-cell maintains responsiveness to exogenously administered GLP-1, but the response may be somewhat attenuated compared with that seen in the non-diabetic state, indicating a partial incretin defect with GLP-1.^9^ Due to these effects on the β-cell, GLP-1 became an attractive pharmacologic target, and harnessing it in some fashion was enticing. This had to be accomplished while overcoming a key shortcoming of the native GLP-1 peptide, namely its susceptibility to rapid breakdown by the ubiquitous protease dipeptidyl peptidase-4 (DPP-4), rendering the native hormone incapable of a useful pharmacologic effect unless infused continuously. Over the past 5 years, we have seen the emergence of the DPP-4 inhibitor oral agent class and the injectable GLP-1 receptor agonist class. Both pharmaceutical preparations have overcome the instability of native GLP-1 and have been important new additions, with well-documented beneficial effects on glycemic control and short-term effects on β-cell function. However, to date, no long-term β-cell-preserving effects have been documented. Regardless, the wider physiologic role of the incretin hormones has uncovered a wide array of pharmacologic effects that may be of particular advantage to patients with T2D.

## Incretin-based therapies: Leveraging the effects of GLP-1

Much of the initial focus of GLP-1 has been its effect on the β-cell. It is known that GLP-1 enhances insulin secretion in a glucose-dependent manner, meaning that insulin secretion is most demonstrable when plasma glucose is abnormally elevated and not seen when plasma glucose is below the normoglycemic range, thereby resulting in low risk of hypoglycemia.^10^ The effect on the β-cell is immediate and robust, namely restoration of first-phase insulin secretion,^10^ a well-characterized defect seen early in T2D and not corrected by other pharmacological interventions. This response, an artifice of an experimental challenge to the β-cell, gains clinical relevance when a similar early robust response of the β-cell to a meal challenge results in a powerful effect, namely the prevention of an abnormal rise in postprandial plasma glucose. In addition, GLP-1 slows gastric emptying and reduces the secretion of glucagon in a glucose-dependent manner, both of which further contribute to the reduction of elevated postprandial plasma glucose.^10^ The effects of GLP-1 are not confined to the postprandial state, because clinical data demonstrate a lowering of abnormally elevated fasting plasma glucose to the normal range when the hormone is infused intravenously; this appears to be β-cell mediated, because insulin secretion increases concomitantly.^10^ The effects of GLP-1 on the β-cell are not confined to a glucose-dependent stimulation of insulin secretion; favorable effects on upstream insulin biosynthesis are also observed^11^ and, over the longer-term, an effect to potentially restore β-cell function and mass has been observed in preclinical models.^12^–^14^ This preclinical finding has raised speculation concerning the potential for a truly disease-modifying impact of GLP-1 pharmacology on T2D and also possibly on Type 1 diabetes.

The wider array of glucoregulatory effects of GLP-1 can be described as β-cell sparing because they reduce the workload of the β-cell by: (i) suppressing the catabolic hormone glucagon, which is abnormally elevated in the diabetic setting, particularly postprandially; (ii) slowing of gastric emptying postprandially, thereby allowing a more timely delivery of nutrients into the portal and then systemic circulation; (iii) suppressing appetite, reducing food intake and therefore the caloric load required to be assimilated in fuel stores; (iv) reducing body weight, likely a consequence of effects on satiety; (v) insulin-sensitizing effects, clearly seen in animal models^15^ but somewhat disputed in humans and, if observed, often considered to be a secondary response to weight loss; (vi) an insulin-independent effect on glucose clearance from the circulation that may be mediated through portal signaling (Table 1).^15^

**Table 1:** Glucoregulatory effects of glucagon-like peptide-1 (GLP-1) and incretin-based therapies

	GLP-1	GLP-1 receptor agonists	DPP-4 inhibitors
Enhanced glucose-dependent insulin secretion	Yes	Yes, including restoration of first-phase insulin response to i.v. glucose	Yes
Suppression of inappropriately elevated glucagon secretion	Yes	Yes	Yes
Regulation of gastric emptying	Yes	Yes	No
Suppression of appetite	Yes	Yes	No
Weight reduction	Yes	Yes	No: neutral effects on weight
Improved β-cell function	Yes*	Yes	Yes

* Glucagon-like peptide-1 (GLP-1) exerts trophic effects on primary islet cultures, *in vivo*β-cell models, and β-cell lines, promoting β-cell proliferation and neogenesis while reducing β-cell apoptosis.^110^–^113^ In addition, GLP-1 also promotes differentiation of non-insulin cells into cells able to secrete insulin.^114^

DPP-4, Dipeptidyl peptidase-4.

Beneficial effects are also seen in the cardiovascular system, including protective actions in the cardiomyocyte at a molecular level and improved cardiac function. In addition, potentially beneficial anti-inflammatory, cardiorenal, and peripheral vasodilatory effects have been reported.^16^–^18^ There is also a body of work that highlights the potential downstream cardiac effects of the DPP-4-cleaved GLP-1 metabolic product GLP-1(9-36).^18^ It is unclear as yet whether GLP-1(9-36) serves an important physiologic role, how its actions are elicited at the receptor level, and how this cleaved product may influence what we observe with GLP-1-based pharmacology. In summary, GLP-1 pharmacology may have potential cardiovascular benefits for patients with T2D and has the potential to exert specific cardiac benefit in certain acute care settings. That said, there is currently only a limited body of supporting evidence from the clinic.

Glucagon-like peptide-1 (GLP-1) has been an attractive pharmacologic target for some time and it has now been harnessed in two very different forms: the DPP-4 inhibitor class and the GLP-1 receptor agonists. The former is represented by the introduction of three oral agents in the US and a fourth available in Europe. The GLP-1 receptor agonist class first emerged as the twice a day injectable exenatide and, since then, efforts have been directed at leveraging GLP-1 actions in a less frequent dosing platform.

## Glucagon-like peptide-1 receptor agonists: Clinical review

Exenatide (exendin-4) was the first available GLP-1 receptor agonist and was originally isolated from the salivary secretion of the *Gila monster*.^19^ Exenatide has approximately 50% amino acid sequence identity to GLP-1, with which it shares many glucoregulatory effects. However, unlike GLP-1, exenatide is resistant to DPP-4 degradation, which gives it a half-life of 2.4 h in the circulation, making it amenable to twice-daily injectable subcutaneous administration.^20^

Early mechanistic work with the exenatide molecule established many of the attributes already seen with GLP-1: glucose-dependent actions on both β- and α-cells,^21^ restoration of first- and second-phase insulin secretion,^21^ slowing of gastric emptying,^22^ satiety effects at mealtime,^23^ and powerful glucose lowering in both the fasting and postprandial states (Table 1).^22^ In the original major long-term clinical trials with exenatide as an adjunct to oral agents, 10 μg exenatide twice daily (b.i.d.) reduced HbA1c (approximately 1%) and body weight (approximately 3 kg) in patients with T2D.^24^–^27^ This was a unique moment in the treatment of T2D because although historic interventions elicited glycemic improvement, this was invariably coupled with weight gain or, at best, weight neutrality. Trial extension data out to 3 years with the completer population showed exenatide elicited sustained reductions in HbA1c (approximately 1%) and body weight (approximately 5 kg).^28^

The efficacy of exenatide has also been compared with basal and biphasic insulin regimens in patients with T2D.^29^–^32^ Comparing 10 μg exenatide b.i.d. with optimally titrated insulin glargine in trials of up to 52 weeks duration,^29^,^30^,^32^ and a further study comparing 10 μg exenatide b.i.d. or insulin lispro 70/30 for 52 weeks,^31^ revealed equivalent reductions in HbA1c between exenatide and insulin in the range of 0.8–1.4%.^29^–^32^ However, the means by which overall glycemic improvements were achieved differed between the two interventions: insulin treatment had a greater lowering effect on fasting plasma glucose, whereas a greater reduction in postprandial glucose excursion was observed with exenatide.^29^–^32^ Also of note, exenatide reduced body weight in all studies compared with the weight gain seen with insulin treatment.^29^–^32^

A long-acting, sustained-release formulation of exenatide is currently in development for use as a once-weekly injection.^33^ This technology consists of unaltered exenatide encased within a biodegradable medical polymer, namely poly(d,l-lactic-co-glycolic acid), that allows gradual, controlled drug delivery over an extended period of time.^34^ Early clinical work has shown that the once-weekly platform elicits a greater effect on fasting plasma glucose and HbA1c (approximately 1.7% lowering) than that seen with exenatide b.i.d., but similar effects on body weight.^33^ In subsequent longer-term clinical trial work, 2 mg exenatide once weekly has consistently resulted in HbA1c lowering in the 1.5–2.0% range, with weight effects similar to those seen with exenatide b.i.d. Studies have compared exenatide once weekly with exenatide b.i.d., maximal dosing of either pioglitazone or sitagliptin, and optimally titrated glargine insulin.^35^–^37^ In each study, exenatide once weekly has established superiority with regard to lowering HbA1c, coupled with reproducible weight reduction. Exenatide once weekly is currently under review with the US Food and Drug Administration (FDA).

Liraglutide is a modified analog of human GLP-1, with an arginine to lysine amino acid substitution (position 34) and the attachment of a glutamic acid residue with palmitic acid (C-16 fatty acid) to an existing lysine residue (position 26). These modifications render liraglutide 97% homologous to human GLP-1, resistant to DPP-4 cleavage and with an increased half-life of 13 h in the circulation, making it suitable for once-daily administration by subcutaneous injection.^38^ In clinical trials, the maximally recommended dose of liraglutide (1.8 mg) reduced HbA1c by 1.0–1.5% and body weight by approximately 2 kg in different trials in which it was compared with metformin, glimepiride, rosiglitazone, or insulin glargine.^39^–^43^ Of note, liraglutide was also compared with exenatide b.i.d. in a recent 26-week trial and was found to have a significant advantage over exenatide b.i.d. in terms of HbA1c lowering, but equivalent effects on body weight.^44^ Liraglutide was recently approved as a once a day GLP-1 agonist at doses of 0.6–1.8 mg/day.^44^

In addition to exenatide b.i.d., exenatide once weekly, and liraglutide, an array of additional GLP-1 receptor agonists are in earlier stages of clinical development.^45^–^51^ Taspoglutide is a highly modified analog of human GLP-1 (93% homology) with two aminoiso-butyric acid substitutions at positions 8 and 35 and a sustained-release formulation, which makes it resistant to degradation by DDP-4 and potentially suitable for once weekly administration.^45^ However, the development of taspoglutide has recently been suspended owing to serious hypersensitivity reactions and gastrointestinal side effects reported in clinical trials. Albiglutide is a GLP-1 genetic dimer fused to human albumin, which confers resistance to DPP-4, making this agent potentially suitable for once weekly administration.^46^–^48^ Lixisenatide (AVE-0010) is a modified exenatide molecule with six C-terminal lysine residues, which confers resistance to degradation by DPP-4 and favors once or twice daily administration.^49^–^51^

## Dipeptidyl peptidase-4 inhibitors: Clinical review

Dipeptidyl peptidase-4 inhibitors differ greatly from the GLP-1 receptor agonists in that they are small molecules, rather than peptides, and thus can be administered orally rather than by subcutaneous injection. They inhibit the action of DPP-4 to cleave GLP-1 to its inactive form and therefore elevate levels of native GLP-1 in the circulation. Dipeptidyl peptidase-4 is a ubiquitous enzyme in the circulation, where, in addition to GLP-1, it cleaves other peptides, such as native GIP; it is inferred that the glucose-lowering effects of this class of drugs are through actions on GLP-1, but perturbation of other peptide systems cannot be excluded.^52^

There are nuances in the pharmacologic characteristics of the drugs within the class (e.g. the first commercially available DPP-4 inhibitor, sitagliptin, binds non-covalently to the enzyme, whereas vildagliptin and saxagliptin bind covalently to DPP-4), which provide each agent with unique reversible binding properties and potential for differences in their pharmacologic action.

The clinical efficacy of the DPP-4 inhibitors is modest compared with that seen with the GLP-1 receptor agonists (Table 1). In long-term clinical trials, sitagliptin reduced HbA1c in the region of 0.6–0.7%, with a modest reduction in fasting plasma glucose.^53^–^59^ Trials of sitagliptin also demonstrated weight neutrality.^53^–^59^ These findings have been reproduced in clinical work with vildagliptin and saxagliptin, and there is evidence that earlier-stage DPP-4 inhibitors in development also have similar effects.^60^–^66^

## Comparison of GLP-1 receptor agonists and DPP-4 inhibitors

Several recent trials have directly compared incretin-based therapies, to some degree addressing questions concerning relative efficacy and safety of short- versus long-acting GLP-1 receptor agonists and DPP-4 inhibitors.

For example, exenatide and sitagliptin were compared in two 2-week cross-over trials.^23^,^67^ Compared with sitagliptin, exenatide improved 2-h postprandial glucose and insulin secretion, reduced the abnormal rise in postprandial glucagon secretion, reduced plasma triglycerides, slowed gastric emptying, and reduced caloric intake.

In another study, exenatide once weekly was compared with sitagliptin over a period of 26 weeks.^36^ Exenatide once weekly reduced HbA1c and body weight by 1.5% and 2.3 kg, respectively, compared with decreases of 0.9% and 1.5 kg, respectively, seen with sitagliptin.^36^ Exenatide once weekly also improved systolic blood pressure, the albumin:creatinine ratio, and B-type natriuretic peptide compared with sitaglitpin, which had no significant effect on these parameters.^36^

Once-daily liraglutide was compared with sitagliptin in another 26-week study.^68^ Liraglutide reduced HbA1c and body weight by 1.5% and 3.4 kg, respectively, compared with decreases of 0.9% and 1.0 kg, respectively, seen with sitagliptin. Liraglutide also improved fasting plasma glucose (but not postprandial glucose), HOMA-B, C-peptide concentrations, the pro-insulin:insulin ratio, and total cholesterol compared with sitagliptin.^68^

On balance, in head-to-head to studies, the GLP-1 receptor agonist class has demonstrated a more potent glycemic-lowering effect than the DPP-4 inhibitors, coupled with weight loss rather than weight neutrality. Mechanistically, it would appear the islet actions and effects on gastric emptying are more potent with the pharmacologic concentrations achieved with the injectable therapies, coupled with effects on satiety and food intake, which likely contribute to the weight loss observed.

## Safety and tolerability of incretin-based therapies

The introduction of new therapeutic approaches and new pharmaceutical entities requires an appropriate assessment of safety as well as efficacy. The safety profile of GLP-1 receptor agonists seems fairly consistent across different platforms, with some exceptions. First and foremost, the most common issues reported with incretin-based therapies are tolerability issues. Nausea is the most commonly reported event, and tends to be a short-lived phenomenon in the early days or weeks of therapy, but there is a minority of patients who have more severe symptoms and will not tolerate therapy. Other gastrointestinal side effects (e.g. diarrhea) are seen less commonly. Among the more established programs, exenatide once weekly has an improved tolerability profile than exenatide b.i.d. and liraglutide seems similar to exenatide b.i.d., although the overall rate of nausea is reported to be greater with exenatide b.i.d.^35^,^44^ Both liraglutide and exenatide b.i.d. require titration steps to mitigate nausea (two steps for liraglutide, one for exenatide b.i.d., compared with none for exenatide once weekly). The DPP-4 inhibitors, unlike the GLP-1 receptor agonists, are well tolerated with respect to gastrointestinal side effects in most patients.

The risk of hypoglycemia is an important consideration for diabetes therapeutics. Because of the very nature of the glucose dependency of the actions of GLP-1 on β- and α-cells, hypoglycemia should be an uncommon event. Mechanistically, this was demonstrated in an elegant stepped hypoglycemic clamp setting during a concomitant infusion of exenatide, where the stimulatory actions on β-cells and suppressing actions on α-cells were over-ridden promptly once plasma glucose dropped below 90 mg/dL,^21^ thus allowing the primary counter-regulatory responses to hypoglycemia (i.e. suppression of insulin secretion from β-cells and a robust glucagon release from α-cells) to occur unabated. These findings were reaffirmed in the clinic in an early Phase 3 trial in which exenatide b.i.d. was added on a background metformin therapy, with no increase in hypoglycemia despite a reduction in HbA1c of approximately 1%.^25^ The lack of an increased risk of hypoglycemia in such a clinical setting has been reproduced with both the longer-acting form of exenatide and liraglutide and is a clear differentiating feature of this injectable therapy, especially when compared with its injectable counterpart, insulin.^35^–^37^,^39^–^44^ However, it should be noted that the addition of GLP-1 receptor agonists to background sulfonylurea therapy increases the risk of hypoglycemia, necessitating titration of concomitant sulfonylurea dosing, although severe hypoglycemic events are extremely uncommon.^24^ The DPP-4 inhibitors have no evidence of increasing the risk of hypoglycemia, much like the peroxisome proliferator-activated receptor γ agonists and metformin, and unlike the β-cell secretagogue agents, the sulfonylureas and the meglinitides.

More serious but less common safety issues have been highlighted that pertain to the pancreas, kidney, thyroid C-cells, and immunogenicity. Pancreatitis first emerged as a potential safety consideration with the first GLP-1 receptor agonist exenatide b.i.d. and then subsequently the first DPP-4 inhibitor sitagliptin. Pancreatitis was not noted during the original regulatory clinical trial work conducted prior to approval for either compound, but instead during post-marketing safety surveillance, when both therapies had been prescribed in a much larger population. In order to understand the significance of these phenomena, a thorough appreciation of the limitations of post-marketing safety surveillance is key, namely an imprecise numerator (i.e. reporter bias, data quality of safety events and confounders) and denominator (i.e. difficulty in capturing overall population drug exposure). It was speculated that pancreatitis may be a condition seen more often in the T2D population (irrespective of treatment) and this has been confirmed by an independent epidemiological assessment, showing that patients with diabetes have a near threefold increased risk of pancreatitis compared with those without diabetes.^69^ Associated epidemiological data have also reported no increased risk of pancreatitis when one examines patients initiated on either sitagliptin, exenatide b.i.d., insulin, or metformin.^70^,^71^ Studies in animal models have also tried to shed light on this safety issue with mixed findings across a variety of models. Although it is too early to examine the post-marketing experience of liraglutide, a small number of cases of pancreatitis were reported during clinical development; consequently, liraglutide has a similar pancreatitis warning to that seen with exenatide.^72^

In addition, clinically relevant doses of liraglutide and exenatide once weekly and toxicological doses of exenatide b.i.d. have been reported to cause thyroid C-cell adenomas and/or carcinomas in rodents during 2-year carcinogenicity studies (with unknown relevance in humans). Data from limited studies suggest that the human and rodent thyroid may respond differently to GLP-1 because human thyroid cells seem to lack or have low levels of GLP-1 receptors,^73^,^74^ but additional data are needed. As a result of thyroid safety concerns, liraglutide received regulatory approval as an adjunct to diet and exercise, but not as a first-line agent.^72^,^75^ The implications of these safety concerns as they relate to other GLP-1 receptor agonist-based therapies are currently unknown. In clinical studies, there have been no reported cases of thyroid cancer during treatment with liraglutide,^39^–^44^ exenatide b.i.d.,^24^–^27^ or exenatide once weekly.^35^–^37^ However, further preclinical studies and a 15-year cancer registry for liraglutide have been initiated.^75^

In general, DPP-4 inhibitors have a relatively clear safety record, with lower reported rates of adverse events than GLP-1 receptor agonists. The incidence of hypoglycemia with DPP-4 inhibitors has been low, but is higher in patients also using sulfonylurea.^76^

Potential safety concerns with DPP-4 inhibitors include post-marketing reports of hypersensitivity reactions, including anaphylaxis, angioedema, rash, urticaria, and exfoliative skin conditions such as Stevens–Johnson syndrome.^76^ It is speculated that these conditions may result from the blockade of GIP degradation, as well as that of other peptides, such as the glucagon secretin family hormones, pancreatic polypeptide proteins, neuropeptides, and chemokines. Moreover, membrane-bound DPP-4, originally identified as CD26, is involved in immune system function and plays a role in cell signaling, the regulation of extracellular matrix binding, and ion transport.^77^

## Incretin-based therapies: Beyond glycemic control

Incretin-based therapies represent an important step forward in the treatment of T2D, addressing, at least in part, a previously neglected aspect of diabetes pathophysiology, namely the incretin defect. Incretin-based therapies demonstrate a capacity to sustain long-term improvements in glycemic control and, with GLP-1 receptor agonists, significant reductions in body weight. Moreover, incretin-based therapies have the potential for disease modification by eliciting fundamental improvements in β-cell function, primarily noted with the GLP-1 receptor agonist class.^30^,^78^

Emerging data, primarily with GLP-1 receptor agonists, again also reports the improvement of several key cardiovascular risk factors, including lipid profiles and blood pressure. In clinical trials, exenatide b.i.d. treatment was associated with significant improvements in lipid profiles, including high-density lipoprotein–cholesterol (HDL-C), triglycerides, and total cholesterol in patients who were treated for more than 3 years in open-label extension studies.^28^ Data from the liraglutide clinical development program also reported significant improvements in total cholesterol, low-density lipoprotein–cholesterol (LDL-C), and triglycerides.^39^–^44^ Moreover, HDL-C, LDL-C, triglycerides, and total cholesterol were reported to improve with up to 2 years of treatment with exenatide once weekly.^79^–^81^

In addition, effects on diastolic and systolic blood pressure have been observed during the exenatide b.i.d., liraglutide, and once-weekly development programs.^79^–^82^ A recent pooled analysis of six exenatide b.i.d. clinical trials of ≥26 weeks duration demonstrated significant reductions in systolic blood pressure with exenatide compared with placebo or insulin.^82^ Similarly, a pooled analysis of patients participating in two exenatide once weekly trials for 52 weeks also demonstrated significant improvements in systolic blood pressure compared with sitagliptin and pioglitazone.^80^ A meta-analysis from six 26-week trials during liraglutide clinical development reported improved endpoint systolic blood pressure.^83^ It is significant that improvements in cardiovascular risk have been reported to occur prior to, or be weakly correlated with, reductions in body weight in analyses of data from exenatide b.i.d. and once weekly, as well as the liraglutide clinical development programs, suggesting direct effects of GLP-1 receptor agonists on blood pressure.^79^–^83^

Although the impact of these improvements in cardiovascular risk factors on eventual events has not yet been demonstrated directly, the cardiovascular risk profile associated with exenatide b.i.d. treatment has been assessed in a meta-analysis of 12 clinical trials of ≤52 weeks duration. Compared with a pooled comparator group, the relative cardiovascular event risk associated with exenatide treatment was 0.80 [95% confidence interval (CI) 0.53–1.22].^84^

Increasingly, liver fat content is being recognized as another cardiovascular risk factor that often accompanies T2D, because 80% of patients with diabetes are thought to have concurrent fatty liver disease.^85^ There is early evidence that GLP-1 receptor agonists have the potential to improve hepatic health,^28^,^79^,^80^ as measured by hepatic enzyme concentrations, with implications for the treatment of non-alcoholic steatohepatitis and non-alcoholic fatty liver disease, particularly in patients with T2D. Up to 3.5 years of treatment with exenatide b.i.d. or 1 year with exenatide once weekly improved alanine transaminase (ALT) and aspartate aminotransferase (AST), measures of hepatic function that are commonly used surrogates of liver inflammation/steatosis.^28^,^79^,^80^ Exenatide b.i.d. treatment has also been demonstrated to reduce liver fat content in individual patients. For example, a case study reported that a male patient who had been treated with exenatide for 44 weeks experienced a reduction in liver fat of 73% that was associated with improvements in liver function (ALT – 26 IU/L and AST – 5 IU/L).^86^ This was accompanied by improvements in blood pressure, LDL-C, HDL-C, triglycerides, and HOMA-IR,^86^ consistent with the emerging link between liver fat/liver function and cardiovascular risk factors.^87^

Because of the observed effects on cardiovascular risk markers in patients with T2D and the demonstration of GLP-1 receptor localization to cardiac and vascular tissues, several investigations have addressed the question of potential direct effects of GLP-1 receptor agonists on the cardiovascular system. Exenatide has been reported to have cardioprotective effects, measured by preserved cardiac function and reduced infarct size in animal models of ischemia–reperfusion injury in pigs and rodents.^18^,^88^,^89^ Liraglutide was reported to have no cardioprotective effect in a similar pig ischemia–reperfusion injury model,^90^ but it did improve survival in mice following induced myocardial infarction, reducing the number of cardiac ruptures and infarct size and improving cardiac output.^91^ Both exenatide and liraglutide have been reported to modulate the expression of cardioprotective genes and to reduce activation of the apoptotic pathway enzyme caspase-3 during ischemic insults.^88^,^91^

Exenatide b.i.d. has been reported to reduce C-reactive protein, a measure of inflammation,^92^,^93^ total adiponectin, and the oxidative stress marker malondialdehyde compared with insulin glargine in studies of ≤52 weeks duration.^93^,^94^ In addition, 52 weeks of exenatide b.i.d. treatment reduced resistin and retinol binding protein-4 (measures of insulin resistance), as well as C-reactive protein concentrations, compared with glibenclamide.^95^

In comparison with GLP-1 receptor agonists, there are significantly less data available describing the effects of DPP-4 inhibitors on cardiovascular risk. In clinical trials, sitagliptin was reported to elevate HDL-C, whereas vildagliptin was reported to lower cholesterol and triglycerides.^58^,^66^ A systematic assessment of cardiovascular risk across eight randomized trials in the saxagliptin clinical development program reported no increased relative risk (RR 0.43; 95% CI 0.23–0.80) of a compound endpoint (cardiovascular death, myocardial infarct, and stroke) compared with placebo, metformin, or glyburide.^96^

Glucagon-like peptide-1 acts through a well-described gut–central nervous system axis to exert central effects on food intake and satiety. Glucagon-like peptide-1 receptors are located in the brain and emerging evidence suggests that GLP-1 may have effects beyond modulating food intake, including modulating sleep patterns,^97^ limiting inflammation,^98^,^99^ and neuroprotection.^100^–^102^ It should be noted that this evidence is from animal models and that no human data are available; consequently, the potential benefits to humans are speculative in nature.

There is strong evidence to indicate that disrupted sleep (reduced sleep duration and/or quality) leads to reduced glucose tolerance, reduced insulin sensitivity, and dysregulation of appetite.^103^ Consequently, disrupted sleep has been reported to be associated with the increase in risk of developing T2D.^104^,^105^ It has been hypothesized that altered gut–brain satiety and insulinotropic signaling may contribute, in part, to these observed metabolic defects. In support of this, preliminary evidence from animal models is consistent with the existence of a link between GLP-1 receptor signaling and circadian/sleep systems. The *clock* mutant mouse, which is deficient in a central circadian regulator and displays disrupted circadian control, is associated not only with symptoms of the metabolic syndrome and diabetes, but also with increased sensitivity to the effects of exenatide on the regulation of food intake and weight loss.^97^ It is possible that effects of exenatide on glucose tolerance and appetite regulation could be associated with an improvement of sleep duration and quality in patients with disordered sleep, with or without diabetes.

Consistent with preclinical studies indicating that exenatide may have protective and/or regenerative effects on the β-cells in the pancreas, it appears GLP-1 receptor agonism may have similar effects in the brain. Glucagon-like peptide-1 receptors are located in the brain, where both GLP-1 and exenatide gain access via the circumventricular organs. In addition, GLP-1 receptor-knockout mice display reduced synaptic plasticity and disordered learning and memory, suggestive of a role for GLP-1 receptors in normal neural function.^106^ Glucagon-like peptide-1 receptor agonism has been shown to have neurotrophic and neuroprotective effects on neuronal cell types, including the promotion of neurite outgrowth in cultured cells,^100^,^101^ protection of cultured neurons from apoptosis induced by trophic factor deprivation,^102^ oxidative insult, amyloid-β (Aβ) peptide exposure, and excitotoxic stimulation.^107^ Furthermore, in animal models of Alzheimer's disease, GLP-1 receptor agonism has been shown to reduce levels of Aβ peptide in the brain and reduce oxidative damage.^108^ In a mouse model of Parkinson's disease [1-methyl-4-phenyl-1,2,3,6-tetrahydropyridine (MPTP)], exendin-4 protected dopaminergic neurons against MPTP-induced neurodegeneration, thereby preserving dopamine levels and improving motor function.^109^ Together, these findings, as well as a growing body of additional data, provide the basis for speculation that GLP-1 receptor agonism may have beneficial effects in patients with neurodegenerative diseases such as Alzheimer's disease and Parkinson's disease.

**Figure 1:**
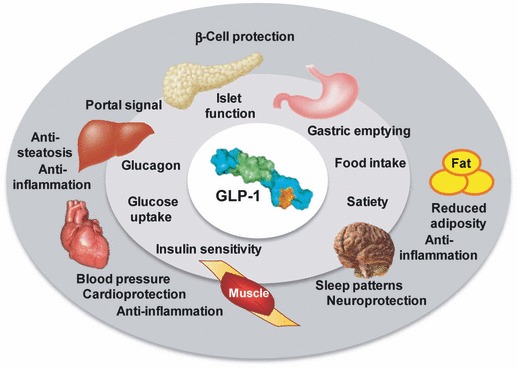
Glucagon-like peptide-1 (GLP-1) has established effects on glycemic control and body weight, and is reported to have positive impacts on cardiovascular risk, inflammation, sleep, and hepatic health. In addition, GLP-1 has been reported to have neuroprotective, neurotrophic, and cardioprotective effects. (See text for details).

The incretin-based therapies are currently labeled for the treatment of patients with T2D. However, the reported improvements in extraglycemic effects of the class render incretin-based therapies, particularly the longer-acting GLP-1 receptor agonists, as potential candidates for the treatment of individuals at risk of not just prediabetes and the metabolic syndrome, where weight and cardiovascular outcomes are key concerns, but also a number of additional disease states, including sleep disorders and neurodegenerative disease (Fig. 1).

## Conclusions

The emergence of the first incretin-based therapies, exenatide and sitagliptin, has impacted the treatment of T2D such that they have become important considerations in the treatment armamentarium. These two pioneer therapies are now followed by additional DDP-4 inhibitor agents and GLP-1 receptor agonists as the incretin-based therapies become increasingly established. Both classes exhibit important glucose-lowering effects and unique positive attributes. Drugs in the DPP-4 inhibitor class are administered orally and exhibit good tolerability and an acceptable safety profile, with the attraction of a low hypoglycemic potential; in addition, they are weight neutral. The GLP-1 receptor agonists are injectable, have short-term gastrointestinal tolerability effects, but appear to have more glucose-lowering potential than the DPP-4 inhibitors. They elicit significant weight loss in many patients and are associated with positive effects on cardiovascular risk factors.

The GLP-1 receptor agonist class holds great promise with the introduction of once-daily therapy (liraglutide) and the possibility of once-weekly and even once- monthly platforms in development. Long-term clinical data are required to determine whether the potential positive effects of incretin-based therapy on β-cell health and the cardiovascular system are fully realized.

## References

[1] Nathan DM, Buse JB, Davidson MB (2009). Medical management of hyperglycemia in T2D: A consensus algorithm for the initiation and adjustment of therapy. A consensus statement of the American Diabetes Association and the European Association for the Study of Diabetes. Diabetes Care.

[2] Perley M, Kipnis DM (1967). Plasma insulin responses to oral and intravenous glucose: Studies in normal and diabetic subjects. J Clin Invest.

[3] Creutzfeldt W (1979). The incretin concept today. Diabetologia.

[4] Nauck MA, Wollschlager D, Werner J (1996). Effects of subcutaneous glucagon–like peptide 1 (GLP–1[7–36 amide]) in patients with NIDDM. Diabetologia.

[5] Kreymann B, Williams G, Ghatei MA, Bloom SR (1987). Glucagon-like peptide–1 7–36: A physiological incretin in man. Lancet.

[6] Holst JJ, Gromada J (2004). Role of incretin hormones in the regulation of insulin secretion in diabetic and nondiabetic humans. Am J Physiol Endocrinol Metab.

[7] Vilsbøll T, Holst JJ (2004). Incretins, insulin secretion and Type 2 diabetes mellitus. Diabetologia.

[8] Nauck MA, Baller B, Meier JJ (2004). Gastric inhibitory polypeptide and glucagon-like peptide-1 in the pathogenesis of T2D. Diabetes.

[9] Toft-Nielsen MB, Damholt MB, Madsbad S (2001). Determinants of the impaired secretion of glucagon-like peptide-1 in type 2 diabetic patients. J Clin Endocrinol Metab.

[10] Zander M, Madsbad S, Madsen JL, Holst JJ (2002). Effect of 6-week course of glucagon-like peptide 1 on glycemic control, insulin sensitivity, and β-cell function in T2D: A parallel-group study. Lancet.

[11] Stein DT, Jayatillake H, Zheng BN (2010). Type 2 diabetic subjects are highly responsive to GLP-1 induced insulin biosynthesis in vivo. Diabetes.

[12] Farilla L, Hui H, Bertolotto C (2002). Glucagon-like peptide-1 promotes islet cell growth and inhibits apoptosis in Zucker diabetic rats. Endocrinology.

[13] Tourrel C, Bailbé D, Meile M-J, Kergoat M, Portha B (2001). Glucagon-like peptide-1 and exendin-4 stimulate β-cell neogenesis in streptozotocin-treated newborn rats resulting in persistently improved glucose homeostasis at adult age. Diabetes.

[14] Tourrel C, Bailbé D, Lacorne M, Meile M-J, Kergoat M, Portha B (2002). Persistent improvement of T2D in the Goto-Kakizaki rat model by expansion of the β-cell mass during the prediabetic period with glucagon-like peptide-1 or exendin-4. Diabetes.

[15] Zheng D, Ionut V, Mooradian V, Stefanovski D, Bergman RN (2009). Exenatide sensitizes insulin-mediated whole-body glucose disposal and promotes uptake of exogenous glucose by the liver. Diabetes.

[16] Nikolaidis LA, Elahi D, Hentosz T (2004). Recombinant glucagon-like peptide-1 increases myocardial glucose uptake and improves left ventricular performance in conscious dogs with pacing-induced dilated cardiomyopathy. Circulation.

[17] Nikolaidis LA, Mankad S, Sokos GG (2004). Effects of glucagon-like peptide-1 in patients with acute myocardial infarction and left ventricular dysfunction after successful reperfusion. Circulation.

[18] Ban K, Noyan-Ashraf MH, Hoefer J, Bolz SS, Drucker DJ, Husain M (2008). Cardioprotective and vasodilatory actions of glucagon-like peptide 1 receptor are mediated through both glucagon-like peptide 1 receptor-dependent and -independent pathways. Circulation.

[19] Eng J, Kleinman WA, Singh L, Singh G, Raufman J-P (1992). Isolation and characterization of exendin-4, an exendin-3 analogue, from *Heloderma suspectum* venom. J Biol Chem.

[20] Kolterman OG, Kim DD, Shen L (2005). Pharmacokinetics, pharmacodynamics, and safety of exenatide in patients with T2D mellitus. J Health Syst Pharm.

[21] Fehse F, Trautmann M, Holst JJ (2005). Exenatide augments first- and second-phase insulin secretion in response to intravenous glucose in subjects with T2D. J Clin Endocrinol Metab.

[22] Kolterman OG, Buse JB, Fineman MS (2003). Synthetic exendin-4 (exenatide) significantly reduces postprandial and fasting plasma glucose in subjects with T2D. J Clin Endocrinol Metab.

[23] DeFronzo RA, Okerson T, Viswanathan P, Guan X, Holcombe JH, MacConell L (2008). Effects of exenatide versus sitagliptin on postprandial glucose, insulin and glucagon secretion, gastric emptying, and caloric intake: A randomized, cross-over study. Curr Med Res Opin.

[24] Buse JB, Henry RR, Han J, Kim DD, Fineman MS, Baron AD, For the exenatide 113 clinical study group (2004). Effects of exenatide (exendin-4) on glycemic control over 30 weeks in sulfonylurea-treated patients with T2D. Diabetes Care.

[25] DeFronzo RA, Ratner RE, Han J, Kim DD, Fineman MS, Baron AD (2005). Effects of exenatide (exendin-4) on glycemic control and weight over 30 weeks in metformin-treated patients with T2D. Diabetes Care.

[26] Kendall DM, Riddle MC, Rosenstock J (2005). Effects of exenatide (exendin-4) on glycemic control over 30 weeks in patients with T2D treated with metformin and a sulfonylurea. Diabetes Care.

[27] Zinman B, Hoogwerf BJ, Durán García S (2007). The effect of adding exenatide to a thiazolidinedione in suboptimally controlled Type 2 diabetes: A randomized trial. Ann Intern Med.

[28] Klonoff DC, Buse JB, Nielsen LL (2008). Exenatide effects on diabetes, obesity, cardiovascular risk factors and hepatic biomarkers in patients with T2D treated for at least 3 years. Curr Med Res Opin.

[29] Heine RJ, Van Gaal LF, Johns D, Mihm MJ, Widel MH, Brodows RG, GWAA study group (2005). Exenatide versus insulin glargine in patients with suboptimally controlled T2D: A randomized trial. Ann Intern Med.

[30] Bunck MC, Diamant M, Cornér A (2009). One-year treatment with exenatide improves β-cell function, compared to insulin glargine, in metformin treated T2D patients: A randomized, controlled trial. Diabetes Care.

[31] Nauck MA, Duran S, Kim D (2007). A comparison of twice daily exenatide and biphasic insulin aspart in patients with T2D who were suboptimally controlled with sulfonylurea and metformin: A non inferiority study. Diabetologia.

[32] Barnett AH, Burger J, Johns D (2007). Tolerability and efficacy of exenatide and titrated insulin glargine in adult patients with T2D previously uncontrolled with metformin or a sulfonylurea: A multinational, randomized, open-label, 2-period, crossover non inferiority trial. Clin Ther.

[33] Kim D, MacConell L, Zhuang D (2007). Effects of once-weekly dosing of a long-acting release formulation of exenatide on glucose control and body weight in subjects with T2D. Diabetes Care.

[34] Tracy MA, Ward KL, Firouzabadian L (1999). Factors affecting the degradation rate of poly(lactide-co-glycolide) microspheres in vivo and in vitro. Biomaterials.

[35] Drucker DJ, Buse JB, Taylor K (2008). Exenatide once weekly versus twice daily for the treatment of T2D: A randomized, open-label, non-inferiority study. Lancet.

[36] Bergenstal RM, Wysham C, MacConell L (2010). Efficacy and safety of exenatide once weekly versus sitagliptin or pioglitazone as an adjunct to metformin for treatment of T2D (DURATION-2): A randomised trial. Lancet.

[37] Diamant M, Van Gaal L, Stranks S (2010). Once weekly exenatide compared with insulin glargine titrated to target in patients with T2D (DURATION-3): An open-label randomised trial. Lancet.

[38] Agersø H, Jensen LB, Elbrønd B, Rolan P, Zdravkovic M (2002). The pharmacokinetics, pharmacodynamics, safety and tolerability of NN2211, a new long-acting GLP-1 derivative, in healthy men. Diabetologia.

[39] Garber A, Henry R, Ratner R (2009). Liraglutide versus glimepiride monotherapy for T2D (LEAD-3 Mono): A randomized, 52-week, phase III, double-blind, parallel-treatment trial. Lancet.

[40] Nauck M, Frid A, Hermansen K (2009). Efficacy and safety comparison of liraglutide, glimepiride, and placebo, all in combination with metformin, in T2D: The LEAD (liraglutide effect and action in diabetes)-2 study. Diabetes Care.

[41] Marre M, Shaw J, Brändle M (2009). Liraglutide, a once-daily human GLP-1 analogue, added to a sulfonylurea over 26 weeks produces greater improvements in glycemic and weight control compared with adding rosiglitazone or placebo in subjects with Type 2 diabetes (LEAD-1 SU). Diabet Med.

[42] Zinman B, Gerich J, Buse JB (2009). Efficacy and safety of the human GLP-1 analog liraglutide in combination with metformin and TZD in patients with T2D mellitus (LEAD-4 Met+TZD). Diabetes Care.

[43] Russell-Jones D, Vaag A, Schmitz O (2009). Liraglutide vs insulin glargine and placebo in combination with metformin and sulfonylurea therapy in T2D mellitus (LEAD-5 met+SU): A randomized controlled trial. Diabetologia.

[44] Buse JB, Rosenstock J, Sesti G (2009). Liraglutide once a day versus exenatide twice a day for T2D: A 26-week randomized, parallel-group, multinational, open-label trial (LEAD-6). Lancet.

[45] Nauck MA, Ratner RE, Kapitza C, Berria R, Boldrin M, Balena R (2009). Treatment with the human once-weekly GLP-1 analogue taspoglutide in combination with metformin improves glycemic control and lowers body weight in patients with T2D mellitus inadequately controlled with metformin alone: A double-blind placebo-controlled study. Diabetes Care.

[46] Matthews JE, Stewart MW, De Boever EH, Albiglutide Study Group (2008). Pharmacodynamics, pharmacokinetics, safety, and tolerability of albiglutide, a long-acting glucagon-like peptide-1 mimetic, in patients with T2D. J Clin Endocrinol Metab.

[47] Bush MA, Matthews JE, De Boever EH (2009). Safety, tolerability, pharmacodynamics and pharmacokinetics of albiglutide, a long-acting glucagon-like peptide-1 mimetic, in healthy subjects. Diabetes Obes Metab.

[48] Rosenstock J, Reusch J, Bush M, Yang F, Stewart M (2009). Potential of albiglutide, a long-acting GLP-1 receptor agonist, in T2D: A randomized controlled trial exploring weekly, biweekly, and monthly dosing. Diabetes Care.

[49] Thorkildsen C, Neve S, Larsen BD, Meier E, Pedersen JS (2003). GLP-like peptide 1 receptor agonist ZP10A increases insulin mRNA expression and prevents diabetic progression in *db/db* mice. J Pharmacol Exp Ther.

[50] Ratner RE, Rosenstock J, Boka G, On behalf of the DRI6012 study investigators (2008). A dose-finding study of the new GLP-1 agonist AVE0010 in T2D insufficiently controlled with metformin. Diabetes.

[51] Christensen M, Knop FK, Holst JJ, Vilsboll T (2009). Lixisenatide, a novel GLP-1 receptor agonist for the treatment of T2D mellitus. IDrugs.

[52] Drucker DJ, Nauck MA (2006). The incretin system: Glucagon-like peptide-1 receptor agonists and dipeptidyl peptidase-4 inhibitors in Type 2 diabetes. Lancet.

[53] Aschner P, Kipnes MS, Lunceford JK, Sanchez M, Mickel C, Williams-Herman DE, Sitagliptin Study 021 Group (2006). Effect of the dipeptidyl peptidase-4 inhibitor sitagliptin as monotherapy on glycemic control in patients with T2D. Diabetes Care.

[54] Raz I, Hanefeld M, Xu L, Caria C, Williams-Herman D, Khatami H, Sitagliptin Study 023 Group (2006). Efficacy and safety of the dipeptidyl peptidase-4 inhibitor sitagliptin as monotherapy in patients with T2D mellitus. Diabetologia.

[55] Charbonnel B, Karasik A, Liu J, Wu M, Meininger G, Sitagliptin Study 020 Group (2006). Efficacy and safety of the dipeptidyl peptidase-4 inhibitor sitagliptin added to ongoing metformin therapy in patients with T2D inadequately controlled with metformin alone. Diabetes Care.

[56] Rosenstock J, Brazg R, Andryuk PJ, Lu K, Stein P, Sitagliptin Study 019 Group (2006). Efficacy and safety of the dipeptidyl peptidase-4 inhibitor sitagliptin added to ongoing pioglitazone therapy in patients with T2D: A 24-week, multicenter, randomized, double-blind, placebo-controlled, parallel-group study. Clin Ther.

[57] Hermansen K, Kipnes M, Luo E, Fanurik D, Khatami H, Stein PP, Sitagliptin Study 035 Group (2007). Efficacy and safety of the dipeptidyl peptidase-4 inhibitor, sitagliptin, in patients with T2D mellitus inadequately controlled on glimepiride alone or on glimepiride and metformin. Diabetes Obes Metab.

[58] Nauck MA, Meininger G, Sheng D, Terranella L, Stein PP, Sitagliptin Study 024 Group (2007). Efficacy and safety of the dipeptidyl peptidase-4 inhibitor, sitagliptin, compared with the sulfonylurea, glipizide, in patients with T2D inadequately controlled on metformin alone: A randomized, double-blind, non-inferiority trial. Diabetes Obes Metab.

[59] Goldstein BJ, Feinglos MN, Lunceford JK, Johnson J, Williams-Herman DE, Sitagliptin 036 Study Group (2007). Effect of initial combination therapy with sitagliptin, a dipeptidyl peptidase-4 inhibitor, and metformin on glycemic control in patients with T2D. Diabetes Care.

[60] Rosenstock J, Aguilar-Salinas C, Klein E, Nepal S, List J, Chen R (2009). Effect of saxagliptin monotherapy in treatment-naïve patients with T2D. Curr Med Res Opin.

[61] DeFronzo RA, Hissa MN, Garber AJ (2009). The efficacy and safety of saxagliptin when added to metformin therapy in patients with inadequately controlled T2D with metformin alone. Diabetes Care.

[62] Hollander P, Li J, Allen E, Chen R (2009). Saxagliptin added to a thiazolidinedione improves glycemic control in patients with T2D and inadequate control on thiazolidinedione alone. J Clin Endocrinol Metab.

[63] Pi-Sunyer FX, Schweizer A, Mills D, Dejager S (2007). Efficacy and tolerability of vildagliptin monotherapy in drug naïve-patients with T2D. Diabetes Res Clin Pract.

[64] Bosi E, Camisasca RP, Collober C, Rochotte E, Garber AJ (2007). Effects of vildagliptin on glucose control over 24 weeks in patients with T2D inadequately controlled with metformin. Diabetes Care.

[65] Bolli G, Dotta F, Rochotte E, Cohen SE (2008). Efficacy and tolerability of vildagliptin vs. pioglitazone when added to metformin: A 24-week, randomized, double-blind study. Diabetes Obes Metab.

[66] Rosenstock J, Baron MA, Dejager S, Mills D, Schweizer A (2007). Comparison of vildagliptin and rosiglitazone monotherapy in patients with T2D: A 24-week, double-blind, randomized trial. Diabetes Care.

[67] Holcombe JH, Shenouda SK, Heilmann CR (2010). Exenatide resulted in significantly greater improvements in 24h average glucose compared to sitagliptin in patients with T2D. Diabetes.

[68] Pratley RE, Nauck N, Bailey T (2010). Liraglutide versus sitagliptin for patients with T2D who did not have adequate glycaemic control with metformin: A 26-week, randomised, parallel-group, open-label trial. Lancet.

[69] Noel RA, Braun DK, Patterson RE, Bloomgren G (2009). Increased risk of acute pancreatitis and biliary disease observed in patients with T2D: A retrospective, cohort study. Diabetes Care.

[70] Bloomgren G, Dore D, Patterson R, Noel R, Braun D, Seeger J (2009). Incidence of acute pancreatitis in exenatide initiators compared to other antidiabetic drug initiators: A retrospective, cohort study. Diabetes.

[71] Herrera V, Aubert R, Tully L (2009). Pancreatitis in patients treated with exenatide or sitagliptin. Diabetes.

[72] http://www.victozapro.com/pdf/Victoza_ComboPI_5.24.pdf.

[73] Bjerre Knudsen L, Madsen LW, Andersen S (2010). Glucagon-like peptide-1 receptor agonists activate rodent thyroid C-cells causing calcitonin release and C-cell proliferation. Endocrinology.

[74] Körner M, Stöckli M, Waser B, Reubi JC (2007). GLP-1 receptor expression in human tumors and human normal tissues: Potential for in vivo targeting. J Nucl Med.

[75] Parks M, Rosebraugh C (2010). Weighing risks and benefits of liraglutide: The FDA's review of a new antidiabetic therapy. N Engl J Med.

[76] http://www.merck.com/product/usa/pi_circulars/j/januvia/januvia_pi.pdf.

[77] McIntosh CHS, Demuth HU, Kim SJ, Pospisilik JA, Pederson RA (2006). Applications of dipeptidyl peptidase IV inhibitors in diabetes mellitus. Int J Biochem Cell Biol.

[78] Bunck MC, Cornér A, Eliasson B (2009). Three-year exenatide therapy, followed by a 4-week off-drug period, had a sustainable effect on β-cell disposition index in metformin treated patients with T2D. Diabetes.

[79] Bergenstal R, Kim T, Yan P (2009). Exenatide once weekly improved cardiometabolic risk factors in subjects with T2D during one year of treatment. Diabetes.

[80] Horton E, Walsh B, Han J (2010). Treatment with once-weekly exenatide for 52 weeks resulted in improvements in glycemic control, body weight, and markers of cardiovascular risk and hepatic injury. Diabetes.

[81] Kim T, Taylor K, Wilhelm K, Trautmann M, Zhuang D, Porter L (2009). Exenatide once weekly treatment elicits sustained glycemic control and weight loss over 2 years. Diabetes.

[82] Okerson T, Yan P, Stonehouse A, Brodows R (2010). Effects of exenatide on systolic blood pressure in subjects with T2D. Am J Hypertens.

[83] Fonseca V, Madsbad S, Falahati A, Zychma MJ, Plutzky J (2009). Once-daily human GLP-1 analog liraglutide reduces systolic BP: A meta-analysis of 6 clinical trials. Diabetes.

[84] Shen L, Han J, Yushmanova I, Bruce S, Porter L (2009). Cardiovascular safety of exenatide BID: An integrated analysis from long-term controlled clinical trials in subjects with T2D. Diabetes.

[85] Bruce KD, Byrne CD (2009). The metabolic syndrome: Common origins of a multifactorial disorder. Postgrad Med J.

[86] Tushuizen ME, Bunck MC, Pouwels PJ, van Waesberghe JH, Diamant M, Heine RJ (2006). Incretin mimetics as a novel therapeutic option for hepatic steatosis. Liver Int.

[87] Gupta NA, Mells J, Dunham RM (2010). Glucagon-like peptide-1 receptor is present on human hepatocytes and has a direct role in decreasing hepatic steatosis in vitro by modulating elements of the insulin signaling pathway. Hepatology.

[88] Timmers L, Henriques JPS, de Kleijn DPV (2009). Exenatide reduces infarct size and improves cardiac function in a porcine model of ischemia and reperfusion injury. J Am Coll Cardiol.

[89] Sonne DP, Engstrøm T, Treiman M (2008). Protective effects of GLP-1 analogues exendin-4 and GLP-1(9-36) amide against ischemia–reperfusion injury in rat heart. Regul Pept.

[90] Kristensen J, Mortensen UM, Schmidt M, Nielsen PH, Nielsen TT, Maeng M (2009). Lack of cardioprotection from subcutaneously and preischemic administered liraglutide in a closed chest porcine ischemia reperfusion model. BMC Cardiovasc Disord.

[91] Noyan-Ashraf MH, Momen MA, Ban K (2009). GLP-1R agonist liraglutide activates cytoprotective pathways and improves outcomes after experimental myocardial infarction in mice. Diabetes.

[92] Kendall D, Bhole D, Guan X (2006). Exenatide treatment for 82 weeks reduced C-reactive protein, HbA1c, and body weight in patients with T2D mellitus. Diabetologia.

[93] Bunck MC, Diamant M, Eliasson B (2009). Beneficial changes on body composition and circulating adiponectin and hsCRP levels following one year of exenatide therapy, compared with insulin glargine, in metformin-treated patients with T2D. Diabetes.

[94] Bunck MC, Cornér A, Eliasson B (2009). One year exenatide therapy, compared with insulin glargine, reduces postprandial oxidative stress in metformin-treated patients with T2D. Diabetes.

[95] Derosa G, Maffioli P, Salvadeo SA (2010). Exenatide versus glibenclamide in patients with diabetes. Diabetes Technol Ther.

[96] Frederich R, Alexander JH, Fiedorek FT (2010). A systematic assessment of cardiovascular outcomes in the saxagliptin drug development program for T2D. Postgrad Med.

[97] Marcheva B, Bass J, Rmasey K (2008). Impact of circadian gene transcription factors in pancreatic β cell function and diabetes. Diabetes.

[98] Varanasi A, Chaudhuri A, Dhindsa S (2011). Durability of effects of exenatide treatment on glycemic control, body weight, systolic blood pressure, C-reactive protein, and triglyceride concentrations. Endocr Pract.

[99] Wu JD, Xu XH, Zhu J (2011). Effect of exenatide on inflammatory and oxidative stress markers in patients with type 2 diabetes mellitus. Diabetes Technol Ther.

[100] Perry T, Holloway HW, Weerasuriya A (2007). Evidence of GLP-1-mediated neuroprotection in an animal model of pyridoxine-induced peripheral sensory neuropathy. Exp Neurol.

[101] Perry T, Lahiri DK, Chen D (2002). A novel neurotrophic property of glucagon-like peptide 1: A promoter of nerve growth factor-mediated differentiation in PC12 cells. J Pharmacol Exp Ther.

[102] Biswas SC, Buteau J, Greene LA (2008). Glucagon-like peptide-1 (GLP-1) diminishes neuronal degeneration and death caused by NGF deprivation by suppressing Bim induction. Neurochem Res.

[103] Barone MT, Menna-Barreto L (2011). Diabetes and sleep: A complex cause-and-effect relationship. Diabetes Res Clin Pract.

[104] Spiegel K, Knutson K, Leproult R, Tasali E, Van Cauter E (2005). Sleep loss: A novel risk factor for insulin resistance and T2D. J Appl Physiol.

[105] Mallon L, Broman JE, Hetta J (2005). High incidence of diabetes in men with sleep complaints or short sleep duration: A 12-year follow-up study of a middle-aged population. Diabetes Care.

[106] Abbas T, Faivre E, Hölscher C (2009). Impairment of synaptic plasticity and memory formation in GLP-1 receptor KO mice: Interaction between type 2 diabetes and Alzheimer's disease. Behav Brain Res.

[107] Perry T, Haughey NJ, Mattson MP, Egan JM, Greig NH (2002). Protection and reversal of excitotoxic neuronal damage by glucagon-like peptide-1 and exendin-4. J Pharmacol Exp Ther.

[108] Perry T, Lahiri DK, Sambamurti K (2003). Glucagon-like peptide-1 decreases endogenous amyloid-β peptide (Aβ) levels and protects hippocampal neurons from death induced by Aβ and iron. J Neurosci Res.

[109] Kim S, Moon M, Park S (2009). Exendin-4 protects dopaminergic neurons by inhibition of microglial activation and matrix metalloproteinase-3 expression in an animal model of Parkinson's disease. J Endocrinol.

[110] Näslund E, Barkeling B, King N (1999). Energy intake and appetite are suppressed by glucagon-like peptide-1 (GLP-1) in obese men. Int J Obes Relat Metab Disord.

[111] Gutzwiller JP, Drewe J, Göke B (1999). Glucagon-like peptide-1 promotes satiety and reduces food intake in patients with diabetes mellitus type 2. Am J Physiol.

[112] Kieffer TJ, Habener JF (1999). The glucagon-like peptides. Endocr Rev.

[113] Zhou J, Wang X, Pineyro MA, Egan JM (1999). Glucagon-like peptide 1 and exendin-4 convert pancreatic AR42J cells into glucagon- and insulin-producing cells. Diabetes.

[114] Perfetti R, Zhou J, Doyle ME, Egan JM (2000). Glucagon-like peptide-1 induces cell proliferation and pancreatic-duodenum homeobox-1 expression and increases endocrine cell mass in the pancreas of old, glucose-intolerant rats. Endocrinology.

